# Acoustic and thermal simulations of tcMRgFUS in patient specific models: validation with experiments

**DOI:** 10.1186/2050-5736-3-S1-P35

**Published:** 2015-06-30

**Authors:** Urvi Vyas, Taylor Webb, Rachelle Bitton, Butts Kim Pauly, Pejman Ghanouni

**Affiliations:** 1 Stanford University, Stanford, California, United States

## Background/introduction

In tcMRgFUS, acoustic and spatial heterogeneities of the skull cause reflection, attenuation, and phase aberrations of the acoustic beams, which may cause patient-specific thermal responses to the same transducer power. In this work, we use acoustic and thermal simulations based on patient-specific 3D heterogeneous tissue models to predict heating at the center of the brain. We validate the model through comparison of the simulated temperature rises to experimentally derived energies for five patients treated using tcMRgFUS. Further, we separate the components of energy loss in the acoustic simulations into reflection-only, attenuation-only, phase aberration-only, and reflection and attenuation both to understand the cause of potential inter-patient variability in these treatments.

## Methods

In five cases, human CT scans were used to create acoustic and thermal tissue models. The hybrid angular spectrum technique[1] was used to model the acoustic beam propagation of the InSightec ExAbalate brain system, for each patient’s skull geometry, yielding maps of the specific absorption rate (SAR). Finite Difference Time Domain simulation of Penne’s Bioheat Transfer Equation were used to model the temperature.

Tissue properties used in the simulations are given in Table 1. Simulated skull efficiency was calculated for each case using the following equation,

Simulated Skull Efficiency=Power23°C temp rise/Powermin.

where Power23C temp rise is the power required in the simulation to reach a 23°C temperature rise in the center of the brain for a 10 second sonication and the Powermin is the minimum power required in the simulation to reach a 23°C temperature rise in the five data sets. Additionally, acoustic simulations for each skull used the tissue property groupings specified in Table 1B-1E to quantify effects of beam propagation. Simulated skull efficiency was compared to experimental energy efficiency calculated for each case using the following equation,

Experimental Energy Efficiency = Energy/Energymin

where Energy was the energy used in the final sonication to reach a temperature of 53-60°C and Energymin was the minimum energy of the final sonication in the group of 5 datasets. These data were derived from five patients who underwent tcMRgFUS treatment conducted using the InSightec ExAbalate 4000 650 kHz brain system.

**Table 1:** Tissue properties[2], voxel size = 0.5x0.5x0.685 mm, calculation time = 80 min, thermal properties[3].

Simulation-Type	Attenuation (Np/cm)	Speed of Sound (m/s)	Density (kg/m3)
A. Skull Efficiency	avoxel = amin + (amax – amin)(1 – Ø) amin = 0.08, amax = 3.2	cvoxel = cmin + (cmax – cmin)(1 – Ø) cmin = 1500, cmax = 2100	dvoxel = Ø x dwater + (1 – Ø)dbone dwater = 1000, dbone = 2100

B. Attenuation-only	avoxel = amin + (amax – amin)(1 – Ø)	Homogeneous = 1550	Homogeneous = 1000

C. Phase Aberration-only	Homogeneous = 0.08	cvoxel = cmin + (cmax – cmin)(1 – Ø)	d’voxel = 1000/cvoxel

D. Reflection-only	Homogeneous = 0.08	Homogeneous = 1550	d’voxel = dvoxel x cvoxel

E. Attenuation and Reflection	avoxel = amin + (amax – amin)(1 – Ø)	Homogeneous = 1550	d’voxel = dvoxel x cvoxel

## Results and conclusions

**Figure 1:**
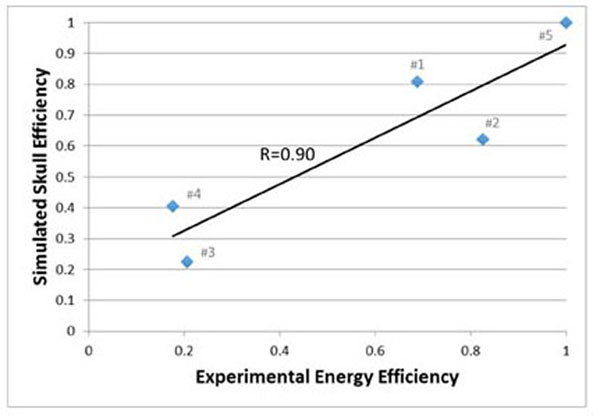
The correlation between the experimental energy efficiency and the simulated skull efficiency is plotted, with a correlation coefficient of 0.90. The treatment case number (correlating with Figure 2) is given in the data labels.

**Figure 2:**
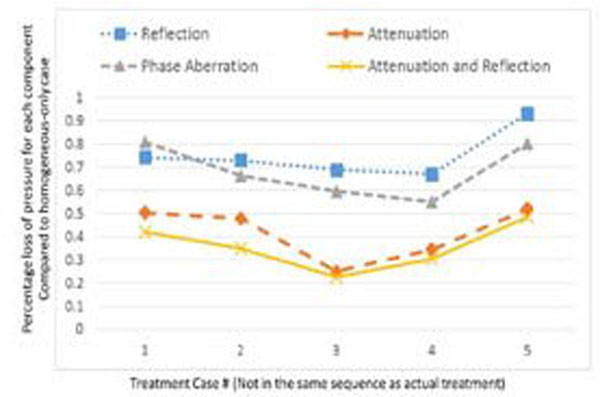
The overall effect of skull heterogeneity is decomposed into the individual components - attenuation, reflection, phase aberration and attenuation and reflection - for each individual skull. The Y-axis plots the loss in focal pressure due to the the component considered, normalized to a homogeneous-only case.

Figure 1 plots the experimental energy efficiency *vs*. the simulated skull efficiency for the five patient’s skulls, showing a correlation of 0.90. Figure 2 decomposes (using simulations) the overall effect of heterogeneity into the individual components - attenuation, reflection, phase aberration and attenuation and reflection - for each skull. The simulated skull efficiency using individual-specific heterogeneous models predicts well (R=0.9) the experimental energy efficiency, while being computationally feasible. Sources of noise in the data include differences in the simulated and the experimental focal location, simulated temperature rise of 60°C *vs*. experimental temperature rise over a range from 53°C-60°C and accuracy of phase correction. The decomposed pressure simulations quantify the role of individual acoustic effects and demonstrates that both reflection and attenuation vary between subjects.
